# Integrated analysis of genome-wide DNA methylation and gene expression profiles in molecular subtypes of breast cancer

**DOI:** 10.1093/nar/gkt643

**Published:** 2013-07-24

**Authors:** Je-Keun Rhee, Kwangsoo Kim, Heejoon Chae, Jared Evans, Pearlly Yan, Byoung-Tak Zhang, Joe Gray, Paul Spellman, Tim H.-M. Huang, Kenneth P. Nephew, Sun Kim

**Affiliations:** ^1^Interdisciplinary Program in Bioinformatics, Seoul National University, Seoul 151-742, Korea, ^2^Bioinformatics Institute, Seoul National University, Seoul 151-744, Korea, ^3^School of Informatics and Computing, Indiana University, Bloomington, IN 47408, USA, ^4^Division of Biomedical Statistics and Informatics, Mayo Clinic, Rochester, MN 55905, USA, ^5^The Ohio State University Comprehensive Cancer Center Nucleic Acid Shared Resource-Illumina Core, Columbus, OH 43210, USA, ^6^School of Computer Science and Engineering, Seoul National University, Seoul 151-742, Korea, ^7^OHSU Knight Cancer Institute, Portland, OR 97239, USA, ^8^Department of Molecular Medicine/Institute of Biotechnology, The University of Texas Health Science Center at San Antonio, San Antonio, TX 78229-3900, USA, ^9^Medical Sciences, Indiana University School of Medicine, Bloomington, IN 47405, USA and ^10^Department of Cellular and Integrative Physiology, Indiana University School of Medicine, Indianapolis, IN 46202, USA

## Abstract

Aberrant DNA methylation of CpG islands, CpG island shores and first exons is known to play a key role in the altered gene expression patterns in all human cancers. To date, a systematic study on the effect of DNA methylation on gene expression using high resolution data has not been reported. In this study, we conducted an integrated analysis of MethylCap-sequencing data and Affymetrix gene expression microarray data for 30 breast cancer cell lines representing different breast tumor phenotypes. As well-developed methods for the integrated analysis do not currently exist, we created a series of four different analysis methods. On the computational side, our goal is to develop methylome data analysis protocols for the integrated analysis of DNA methylation and gene expression data on the genome scale. On the cancer biology side, we present comprehensive genome-wide methylome analysis results for differentially methylated regions and their potential effect on gene expression in 30 breast cancer cell lines representing three molecular phenotypes, luminal, basal A and basal B. Our integrated analysis demonstrates that methylation status of different genomic regions may play a key role in establishing transcriptional patterns in molecular subtypes of human breast cancer.

## INTRODUCTION

The addition of a methyl group to cytosine residues in the context of CpG dinucleotides (i.e. 5-methylcytosine) by the DNA methyltransferease enzymes is the most well-studied epigenetic event. DNA methylation is known to play significant roles in many cellular processes, including embryonic development, genomic imprinting, X-chromosome inactivation and preservation of chromosome stability. In addition, aberrant DNA methylation has been shown to disrupt many cellular processes and is frequently observed in most human diseases, including cancer (1–4).

Methylation in CpG islands (CGIs), particularly in the promoter and first exon regions, is known to block genomic binding sites of activating transcription factors (TFs) or other proteins, and it is strongly associated with gene repression (1,5). In particular, the effect of DNA methylation on tumor suppressor genes (TSGs) has been extensively studied (6). Transcriptional silencing of this key class of genes could contribute to defective regulatory processes in cancer, and the promoter CGI hypermethylation of TSG has been observed in a various types of cancers (7,8). However, few studies have examined the complex relationship between DNA methylation and gene expression on a genome-wide scale using accurate high-resolution DNA methylation data.

Profiling of methylated CpG sequences is now possible by using next-generation sequencing technologies, and a number of recent studies have used high-throughput approaches to study DNA methylation (9,10). Although generating enormous amounts (terabytes) of data is possible, single base pair resolution of bisulfite-converted DNA is still costly and highly labor intensive. Recently, cost effective genome-wide methylation approaches that do not rely on bisulfite-treated DNA have been developed, including methylation-sensitive restriction enzymes approaches (11). One approach, the methylated-CpG island recovery assay (12) followed by sequencing, uses methylated-CpG-binding protein complexes with high affinity to methylated CpG dinucleotides in genomic DNA. Now, a technique known as methyl-CpG binding domain-based capture (MBDCap)-seq (13) is able to use double-stranded DNA, does not depend on the application of methylation-sensitive restriction enzymes and generates DNA sequence variation data (14).

## Motivation and research goals

The availability of high-resolution DNA methylation and gene expression data on a genome scale now allows scientists to investigate the functional consequence of DNA methylation in various genomic regions, including CGIs, which have been extensively investigated in the literature (15–17). CGIs are often found near the promoter regions of genes, and the CGI hypermethylation is known to have significant inhibitory effect on gene expression. In normal cells, CGIs are protected from methylation. However, hypermethylation of promoter CGIs of important genes, i.e. TSGs, is frequently observed in cancer cells (18). In addition to CGIs, recent studies have reported that DNA methylation of other genomic regions can alter downstream gene expression. It was recently reported that methylation of CGIs near transcription start sites (TSSs) of genes (18) or in CGI shores (19), regions ∼2 kb outside of CGIs, were both strongly associated with gene expression. In addition, a strong correlation between methylation in the first exon and expression of the corresponding genes was demonstrated (20). Although these recent studies have clearly shown an association between DNA methylation at various genomic regions and gene expression, several questions remain to be answered. Specifically, in our study on the breast cancer cells, research questions are as follows: How does DNA methylation in the different genomic regions contribute to gene expression? Are there subtype specific DNA methylation-gene expression patterns in breast cancer? Does the methylation of TF-binding sites (TFBS) impact TF binding and subsequent gene expression?

To answer these questions, we used genome-wide profiling data from 30 breast cancer cell lines from the Integrated Cancer Biology Program (ICBP, http://icbp.nci.nih.gov/). We integrated MBDCap-seq methylation data and Affymetrix microarray gene expression data (21). The important goals of our study were as follows:

1. Genomic studies have established major breast cancer intrinsic subtypes that show significant differences in incidence, survival and response to therapy (22). Basal-like breast tumors display aggressive clinical behavior and belong to the high-risk breast cancers that typically carry the poorest prognoses (23,24). To investigate whether phenotype specific methylation and expression patterns exist in the basal A, basal B and luminal breast cancer molecular subtypes, we used an information-theoretic approach to identify genes with differentially methylated DNA regions and differential expression levels.
2. To perform an integrated analysis of DNA methylation and gene expression data on a genome-wide scale and to detect subtype-specific effects of DNA methylation in breast cancer cells. We examined relationships between DNA methylation and gene expression using step-wise analysis starting from genes whose expression was significantly altered in a particular subtype.
3. We used Pearson’s correlation analysis and decision tree learning to investigate the effect of DNA methylation in various regions (CGIs, CGI shores, promoter regions, first exons, first introns and second exons) on the breast cancer subtype differential gene expression.
4. To investigate relationship between TFs and DNA methylation in promoter regions, we examined the relationship between DNA methylation specifically at TFBSs and gene expression in the breast cancer molecular subtypes.

## MATERIALS AND METHODS

### Data

We prepared methylation and gene expression data from 30 breast cancer cell lines representing three tumor phenotypes found in patients (21): basal A, basal B and luminal subtypes. Among 30 cell lines, 17 were basal-like and 13 were luminal-like subtypes (Table 1). The basal-like 17 cell lines were further subdivided into 7 basal A and 10 basal B subtypes.

**Table 1.** Thirty breast cancer cell lines and molecular subtypes

Cell line	Subtype
AU565	Lu
BT549	BaB
HCC1569	BaA
HCC1937	BaA
HCC1143	BaA
HCC1428	Lu
HCC202	Lu
MDAMB436	BaB
SUM185PE	Lu
600MPE	Lu
HCC1500	BaB
MDAMB231	BaB
SUM225CWN	BaA
SKBR3	Lu
MDAMB453	Lu
SUM1315MO2	BaB
SUM52PE	Lu
HSS78T	BaB
MCF12A	BaB
MDAMB157VII	Lu
HCC70	BaA
HCC1954	BaA
SUM149PT	BaB
GCC2185	Lu
LY2	Lu
MCF7	Lu
BT20	BaA
MCF10A	BaB
BT474	Lu
SUM159PT	BaB

Lu, luminal; BaA, basal A; BaB, basal B.

Gene expression data from Affymetrix microarray experiments (21) was downloaded. Genome-wide methylation profiles were measured using the MBDcap-seq technique. The double-stranded methylated fragments were sequenced, and reads were mapped to the human reference genome. Methylation levels were calculated by measuring the density of the read coverage (25), as we have described previously.

The microarray gene expression data were processed and analyzed using R and Bioconductor. The expression values were centered by mean-adjusting each log abundance value (subtracting each value from the mean expression value in the cell line).

### Profiling of DNA methylation patterns

To investigate DNA methylation characteristics across the 30 breast cancer cell genomes, methylation profiles were measured on ±10 kb genomic regions around the TSS of each gene. We divided the genomic regions into bins with a size of 100 bases. DNA methylation levels were then measured as the number of mapped reads within each bin.

### Identifying differentially methylated/expressed genes by information theoretic analysis

We identified differentially methylated and expressed genes in the three breast cancer subtypes using normalized entropy. Entropy is a measure of uncertainty, defined as follows:

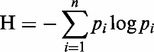

where *p_i_* denotes the probability of the state *i*, and *n* is the total number of the states. In this study, the state *i* is a cancer phenotype, i.e. 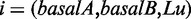. For methylation profiles, the probability *p_i_* is measured by 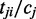, where *c_j_* is sum of read counts for cell lines in a genomic region *j* and *t_ji_* is sum of reads for a phenotype *i* in the region *j*. For gene expression, *c_j_* is sum of expression values for cell lines in a gene *j*, and *t_ji_* is sum of expression for a phenotype *i* in the gene *j*. The entropy *H* achieves its maximum value when all states are equally probable, that is, it exhibits the lowest degree of uncertainty. If there is only one state, then the entropy *H* is zero.

Normalized entropy is the ratio of entropy to maximum entropy as follows:

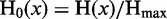

where *H_max_* is maximum entropy value where the probabilities are all equal.

We measured the normalized entropy and identified differentially methylated regions and differentially expressed genes. To avoid errors on the probability calculation, we introduced pseudo-probability to every zero-valued position.

### Identifying downregulated genes in each subtype for integrative analysis

Genes differentially expressed in each different molecular subtype were further identified as follows. Suppose that *e_gl_* is an expression level of a gene *g* in a cell line *l*. As the cell line *l* is clustered into a specific subtype *i*, we calculate the median values 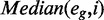 for the expression levels in each subtype *i* per gene *g*. In this study, we measured three median value 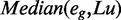, 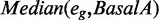 and 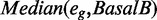 for each gene *g*.

If the median value 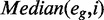 of a gene *g* in a type *i* was significantly lower than those of other two types, we defined the gene *g* as downregulated in a specific type. In our study, log-ratio 1.5 was the criterion for significance.

### Correlation between DNA methylation and gene expression

**Figure 1.**
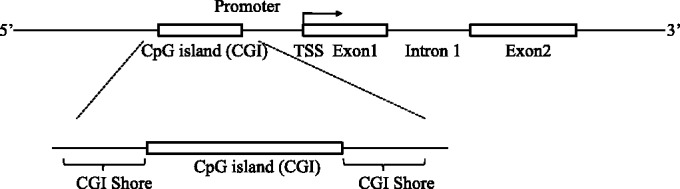
Genomic regions for studying DNA methylation profiles. A gene body is composed of promoter and coding regions including exons and introns. CGIs as well as these regions were studied for the effect of DNA methylation on gene regulation.

To investigate the relationship between methylation in various regions and gene expression in the 30 breast cancer cells, we examined methylation levels in gene promoter regions (2 kb upstream regions from TSSs), CGIs, CGI shores, the first and second exon and the first intron (Figure 1).The association between gene expression and methylation values of these data sets was measured by a Pearson’s correlation coefficient. It was calculated on the paired data of a gene expression level and the methylation level in the genomic region.

### Combinatorial effects of DNA methylation in various genomic regions

To identify which regions have dominant effects on downstream gene expression and also to investigate on the combinatorial roles of DNA methylation of the various genomic regions in each subtype, a decision tree was constructed using the methylation profiles in each region. For the learning purpose, a gene was an instance of data, and gene expression was considered as a class variable, i.e. ‘up- or downregulated genes’. The methylation value in each genomic region was an attribute. For binary classification, in training data set of each subtype, the class values were discretized to high and low, i.e. ‘upregulated or downregulated genes’. If a gene was significantly downregulated in a subtype, but the gene was upregulated in the other subtypes, the class values of the genes in the cell lines within the subtype were designated as low. For example, assume that the expression of a gene is significantly downregulated in Lu subtype. Then among 30 cell lines, 13 instances with Lu subtype are marked as low and 17 with the other types are high. The trees were built using REPTree in WEKA software (26).

### Analysis of TF-binding regions possibly blocked by DNA methylation

For the integrative analysis of TFs, DNA methylation and gene expression, we used four data sets: gene expression, methylation profiles, cell specific DNA sequences and information for TFBSs [TRANSFAC database (27)]. We considered only downregulated genes in each subtype, as we were most interested in DNA methylation of TFBSs, possible interference on TF binding, and subsequent negative effect on gene expression. We referred to these downregulated as ‘target genes’. Differentially methylated genomic regions of the target genes were identified by statistical testing (*t*-test) of methylation levels at each 100 bp bin for the promotor regions. Cell-specific consensus sequences were computed by assembling short reads in the promotor regions of these genes. TFBSs were searched on the cell-specific consensus sequences corresponding to the hypermethylated bins, using ‘minimize false positive’ option of the match tool in the TRANSFAC package (28).

Among the collected TFs that could be potentially blocked by TFBS methylation in the promotor region, we selected TFs whose expression levels were not significantly different in each phenotype (by *t*-test), as to exclude cases where the downregulation of the target genes is as a result by difference in the expression levels of TF, an activator gene. In this way, we compiled cases where downregulation of the target genes was due only to the hypermethylation in the promotor region, not other factors, such as the genomic sequences on the TFBSs and the expression levels of the TF.

## RESULTS

### DNA methylation in 30 ICBP cell lines

**Figure 2.**
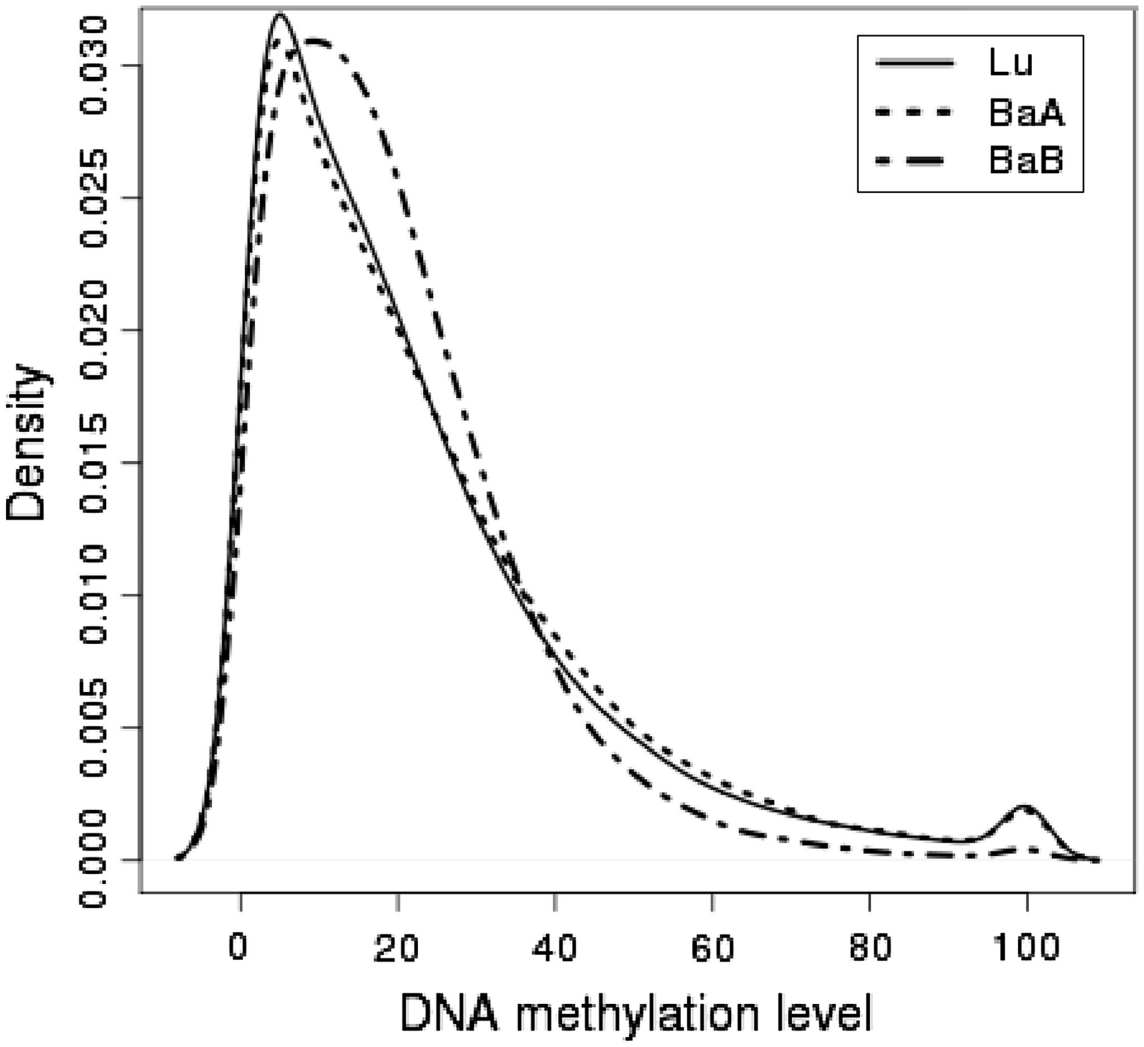
Methylation density of promoter regions in 30 breast cancer cell lines. Density was measured for each subtype. The methylation levels are on the *x*-axis, and the *y*-axis is probabilistic density. Unusual bulbs around 100 on the *x*-axis were because methylation levels over 100 were truncated to 100. Lu, luminal; BaA, basal A; BaB, basal B.

We measured and compared the methylation density of 2 kb promoter regions for all genes in 30 breast cancer cell lines. Figure 2 shows subtype-specific density plots of promoter regions, excluding unmethylated genes. Overall, the methylation density was similar in each subtype. We observe that the number of highly methylated (>50) promoter regions tended to be lower in basal B (BaB). The density of the regions whose methylation levels were over 50 was around 10% in Lu and basal A (BaA), but 4% in BaB.

Next, we investigated CGI methylation around each gene. CGIs are defined as regions of DNA of greater than 500 bp with a G + C equal to or greater than 55% and observed CpG/expected CpG of 0.65 (29). Using the position information of the CGIs from UCSC genome browser, we checked the methylation profile in the CGI near each gene. In the 30 breast cancer cell lines, ∼47% of CGIs were methylated; however, distinct methylation density for each subtype was not apparent (Supplementary Figure S1).

### Analysis protocol 1: information theoretic analysis of phenotype-differentially methylated and expressed genes

To identify differentially methylated and expressed genes across the breast cancer genome, we measured normalized entropy. Lower entropy corresponded to genes more differentially methylated or expressed in each subtype. First, we determined which genes were differentially methylated. Considering only genes with >3 mapped reads, there were 241 differentially methylated genes with the entropy threshold 0.2 and 564 differentially expressed genes with entropy threshold 0.5. Among these, only three genes were common to both the differentially methylated and expressed gene sets (Supplementary Table S1). Thus, we concluded that separate analysis of differentially methylated and expressed gene sets based on information theory is not effective for the integrated analysis of methylation and gene expression, although these methods were effective to highlight genes and genomic regions that were different according to phenotypes.

### Analysis protocol 2: integrated analysis of DNA methylation and gene expression

To perform the integrated analysis of DNA methylation and gene expression, we used a two-step analysis process: (i) identify differentially expressed genes in each subtype and (ii) for each genomic region, test whether there is a strong negative correlation between methylation level at the genomic region and the expression level of the gene.

To select differentially expressed genes in each subtype, we measured median values of expression levels for each of the three breast cancer phenotypes. If the median value of a gene in one subtype was significantly higher or lower than the median value in the other two subtypes, the gene was considered to be differentially expressed in a specific type. For such differentially expressed genes, variations of methylation levels were then investigated.

**Figure 3.**
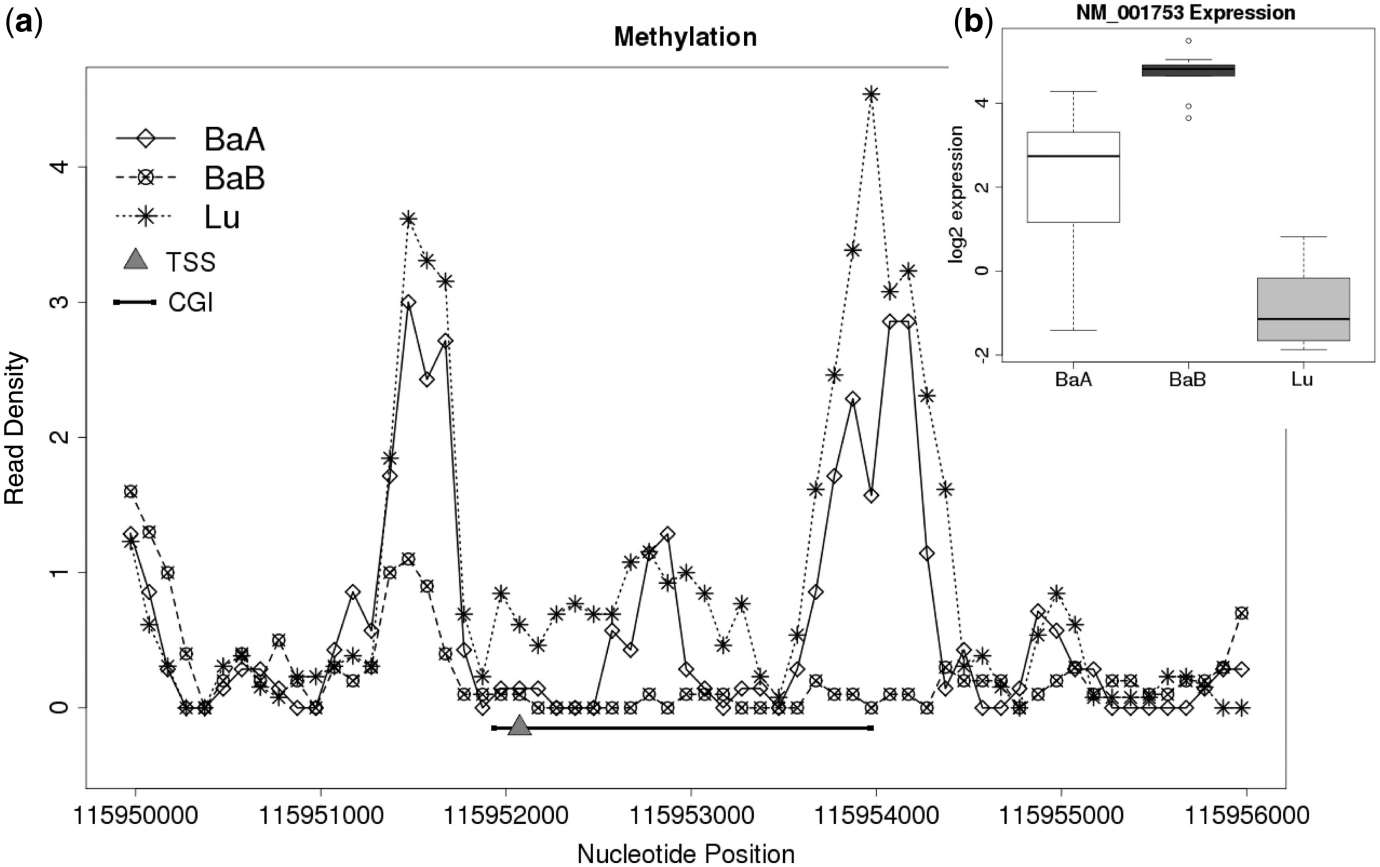
CGI methylation and gene expression of the CAV1 gene. Methylation and gene expression values from the 30 cell lines are grouped into luminal (Lu), BaA and BaB subtypes. (**a**) A plot showing the density of methylation in the CGI and shore regions located near the TSS of the CAV1 gene. The black bar shows the location of the CGI and the small orange triangle is the TSS. (**b**) A boxplot showing the expression of the CAV1 gene.

**Figure 4.**
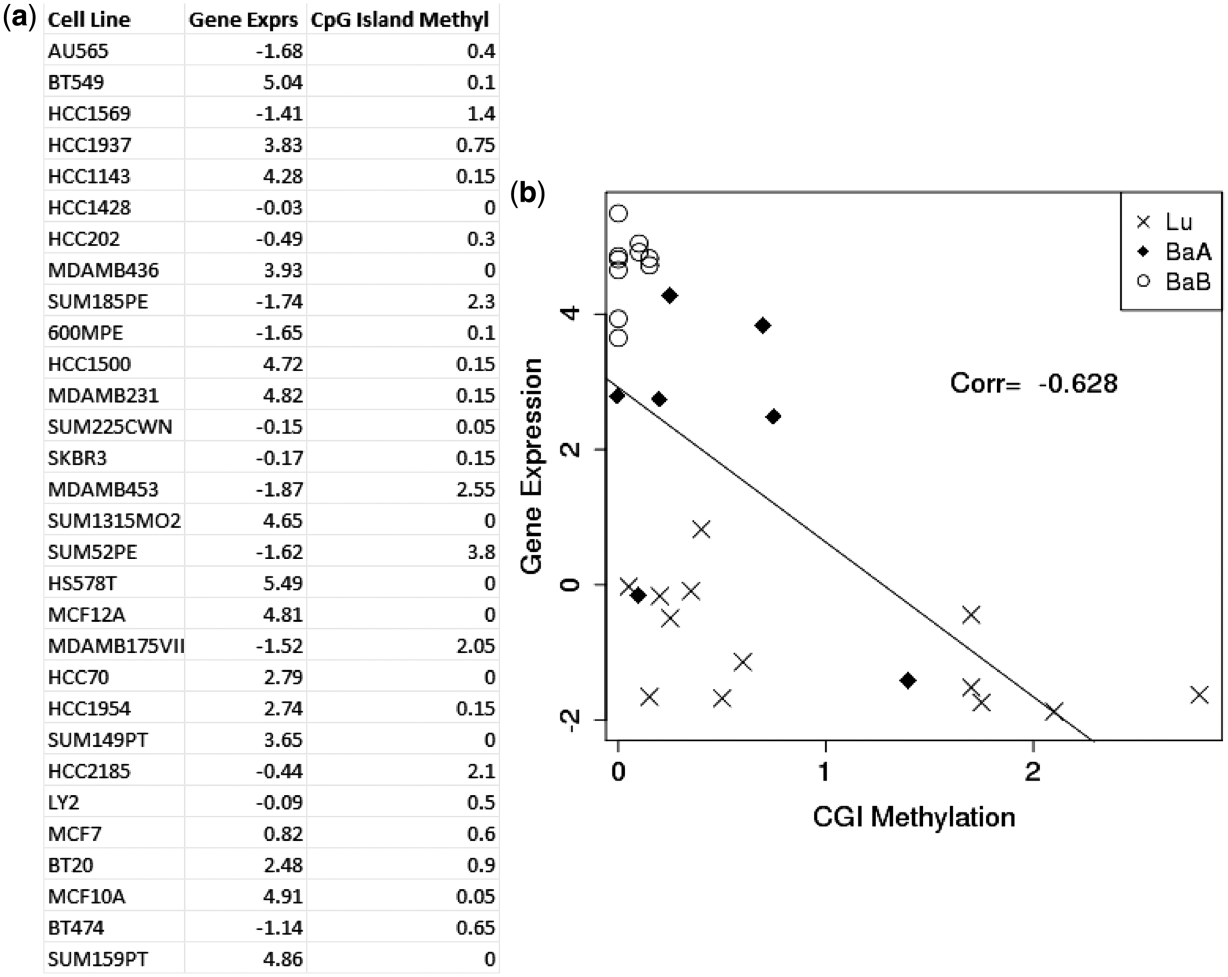
An example of the paired input data used to measure the Pearson correlation between gene expression and methylation. This paired data are for CAV1 gene. (**a**) Gene expression and CGI methylation across 30 cell lines. (**b**) Plot of gene expression profiles (*y*-axis) versus methylation levels (*x*-axis). Each pair in the cells is represented as a cross sign (Lu), a diamond (BaA) and a circle (BaB). A regression line is shown.

As DNA methylation is known to inhibit gene expression and an inverse correlation between the DNA methylation and gene expression has been shown to exist, we were most interested in a negative correlation between DNA methylation and gene expression for the integrated analysis. As an example, Caveolin 1, *CAV1*, represents a negative relationship between DNA methylation and gene expression (Figure 3). The *CAV1* gene has been shown by us and others to regulate breast tumor growth and metastasis and is overexpressed in basal-like subtypes (25,30,31). *CAV1* expression levels were clearly different in each breast cancer subtype, higher in BaB subtypes and lower in Lu subtypes. However, when the DNA methylation profiles of the *CAV1* TSS and CGI were examined, methylation levels were significantly higher in the Lu compared with BaA and BaB. Furthermore, differential methylation of CGI shores, but not CGIs, significantly regulated *CAV1* expression, and breast cancer aggressiveness was associated with *CAV1* CGI shore methylation levels (25). The aforementioned negative correlation was measured by computing Pearson correlation coefficients. The Pearson correlation is measured by paired input data between DNA methylation profiles and gene expression levels across the 30 breast cancer cell lines. As an example, a correlation coefficient from CGI methylation and gene expression levels was calculated across 30 cell lines (Figure 4). The scatter plot for *CAV1* gene shows that gene expression and CGI methylation levels were negatively correlated. The similar trends between DNA methylation and gene expression were observed in many other genes (Supplementary Figure S2).

**Figure 5.**
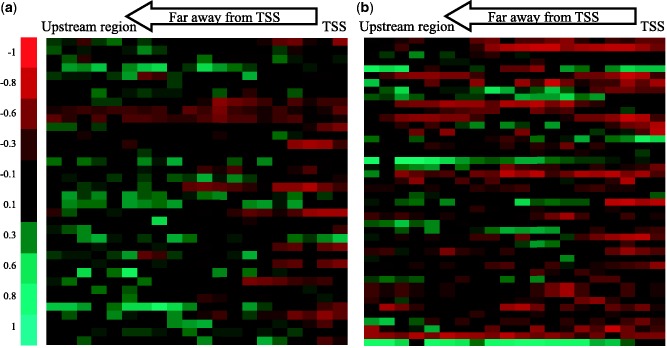
Correlation between promoter region methylation profiles and expression levels of genes downregulated in (**a**) Lu and (**b**) BaB subtypes. Unmethylated genes in the whole promoter region of 30 cell lines were excluded. Light red color was used for negative correlation and light green for positive correlation. Columns from right to left denote positions getting away from TSS. Each row is a downregulated gene in the subtype.

**Figure 6.**
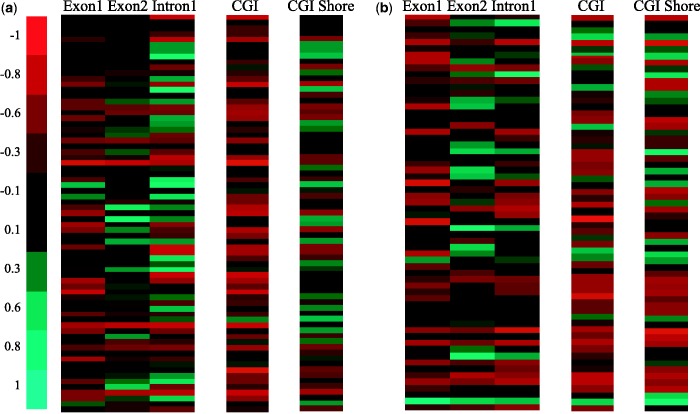
Correlation between methylation profiles on CGI, CGI shore, intron and exon regions and expression levels of genes down-regulated in (**a**) Lu subtypes and (**b**) BaB subtypes.

We measured the methylation correlation for various genomic regions of downregulated genes in Lu and BaB subtype (Figures 5 and 6). As only two genes were detected as downregulated in BaA subtype, the correlation results for BaA subtype were not included. Interestingly, when methylation in promoter regions was considered, several genes showed a clear negative correlation at the proximal regions of TSSs. Figure 5 is heatmaps that visualize promoter region methylation and downstream gene expression (light red colors mean that two vectors (methylation profiles and expression levels) were highly negatively correlated and bright green were positively correlated), and the gene at each row is provided in Supplementary Tables S2 and S3. In both Lu and BaB subtypes, strong negative correlations were observed in promotor regions, and methylation in the promotor regions near TSS showed strongest negative correlations. However, there were significant differences in promotor methylation patterns in Lu and BaB subtypes. In Lu subtypes, weaker negative correlations were observed at genomic regions further away from TSS. On the contrary, in BaB subtypes, consistently strong negative correlations were observed in entire promotor regions. Supplementary Table S4 shows the difference of the correlation coefficient in each promoter region, measured by *t*-test. This result implies that the DNA methylation on the promoter region has stronger epigenetic inactivation in Basal-like subtypes and the methylation of this region may contribute to breast cancer progression.

Moreover, in most genes, first exon and CGI methylation levels were negatively correlated with expression levels (Figure 6, Supplementary Tables S5 and S6). From the multi-exon genes, we measured correlation coefficients between the DNA methylation profiles for each exon and intron, and the expression level of the corresponding gene. A clear negative correlation was observed in the first exon, but this was not the case for second exons and first introns, a result consistent with a previous study showing that first exon methylation was closely associated with low gene expression (20). When we examined CGIs and CGI shore regions, negative patterns were also apparent. CGI and CGI shore DNA methylation levels were negatively correlated with gene expression levels in most genes, but in CGIs, much stronger relationships were shown in our data sets.

### Analysis protocol 3: investigation of the combinatorial effects of DNA methylation in various regions on downstream gene expression levels

As DNA methylation occurs in many genomic regions, it was of interest to examine the effect of the various regions on downstream gene expression, particularly which regions may have a dominant effect on gene expression and whether the effects of the regions were similar in each subtype. Toward this goal, we performed a comprehensive study using six distinct genomic regions: promoter regions, CGIs, CGI shores, first and second exons and first introns. Using the DNA methylation profiles in these regions, we performed a machine-learning analysis.

The decision tree is a classification method that uses conjunctions of features for predicting target values in a tree-like hierarchical decision process. As decision tree learning identifies the most informative attributes for classification, this approach was used to discover regions with dominant and combinatorial effects on expression levels. We normalized the methylation levels of each region in a gene by adjusting the scale, then carried out the decision tree analysis.

**Figure 7.**
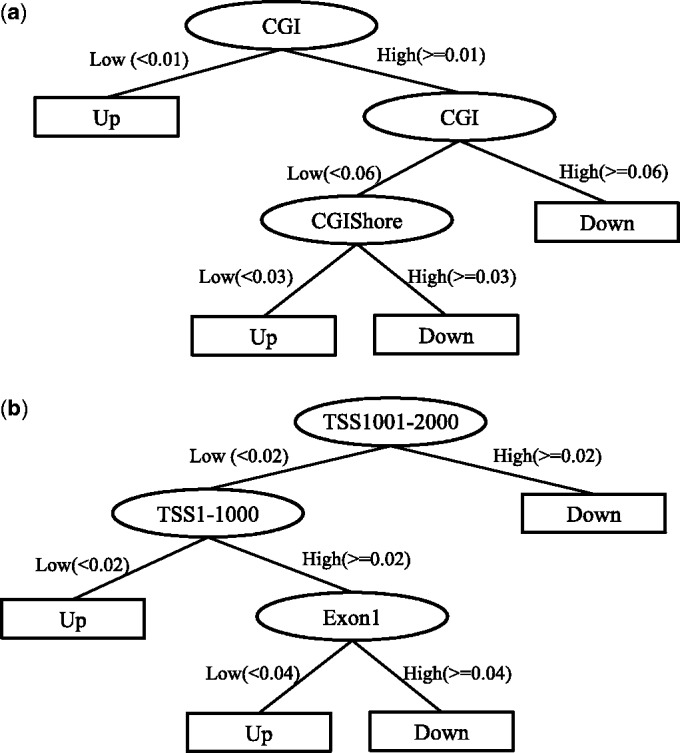
Decision tree analysis with downregulated genes in (**a**) Lu subtypes and (**b**) in BaB. The attributes are represented by circles, in where Exon1 is the first exon and CGIShore means 2 kb outside region from CGI. TSS1-1000 means 1–1000 bp upstream region from TSS and TSS1001-2000 means 1001–2000 bp upstream. The Down in leaf nodes (rectangular boxes) means the gene is downregulated and Up means upregulated.

The decision tree was constructed with a constraint of a maximum tree depth of three excluding leaf nodes, and, in this case, the classification accuracy for genes, downregulated in Lu subtype, was 0.649 in a 10-fold cross-validation (Figure 7a). In the decision tree, the right-most branch means that the nodes in this branch were hypermethylated, and the left-most that the regions were hypomethylated. Consistent with the correlation analysis, CGIs were the most informative feature.

In the BaB subtype whose classification accuracy was 0.746 with the same maximum depth, the promoter regions and the first exons had combinatorial effects on gene expression (Figure 7b). In the left branch of the decision tree where TSS1001-2000 were hypomethylated, it is intuitive that genes were unregulated. However, in the left branch, when TSS1-1000 was hypermethylated and also the first exons were hypermethylated, genes were downregulated. TSS1001-2000 region had the dominant effect on the gene expression in the BaB subtype. This was consistent with our previous correlation analysis showing a clear negative correlation in much broader regions (Figure 5). As CGI overlaps the first exon or promoter regions, we carried out the analysis again by separating into two cases: (i) CGI overlaps with the regions and (ii) CGI does not overlap with the regions. Even when we separated CGI overlapping cases, the dominant factors (CGI for the Lu subtype and TSS1001-2000 for the BaB subtype) remained the same as when we did not separate CGI overlapping cases. The decision trees when we did not separate CGI overlapping cases were presented in the main text (Figure 7), and the decision trees when we separated CGI overlapping cases were presented in Supplementary Figures S3 and S4. The decision tree results suggest that altered gene expression in the two subtypes is associated with not only different promoter methylation profiles but also different combinatorial effects in various genomic regions.

### Analysis protocol 4: integrative analysis of TFs, DNA methylation and gene expression

We next sought to investigate the effect of DNA methylation on the interaction between TF and DNA, i.e. binding of a TF to the promotor region of a gene. To investigate this important concept, we developed a rigorous data mining protocol to compile a list of TF that are potentially blocked by DNA methylation. The schematic overview of the protocol is illustrated in Supplementary Figure S5.

**Figure 8.**
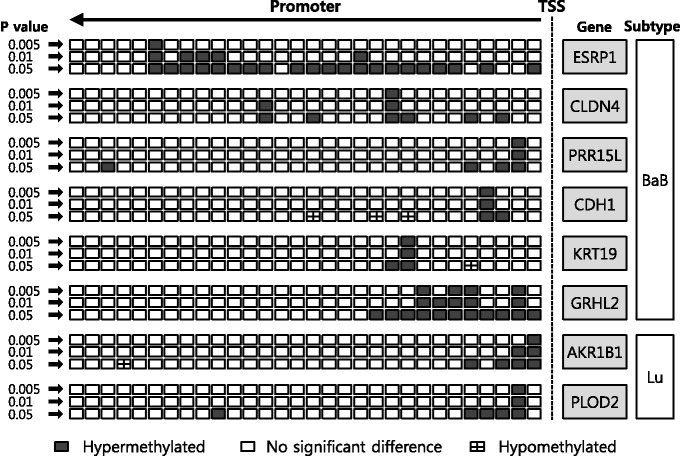
Differentially methylated promoter regions of downregulated genes. Each rectangle in the upstream region means a 100 bp bin.

We first identified differentially methylated genes among the downregulated genes, 60 genes in BaB subtype and 52 genes in Lu subtype. Based on the results of the one side standard *t*-test with a criterion for being significant as *P* < 0.005, we observed eight genes with significant hypermethylation in at least one 100 bp in as follows: *CDH1*, *CLDN4*, *ESRP1*, *GRHL2*, *KRT19*, *PRR15L*, *AKR1B1* and *PLOD2*. Figure 8 shows the promotor regions of the eight genes that are differentially methylated according to the *P*-values.

Next, for the hypermethylated regions of the eight genes, we generated cell line-specific consensus sequences by assembling short reads mapped to the regions and searched candidate TFs, which can be bound to these consensus sequences by match tool (28) on the consensus sequences. To exclude the possibility that higher expression of an activator gene might result in upregulation of target genes, we discarded TFs whose expression levels were significantly different across cell lines of different phenotypes.

Table 2 summarizes the final selection of TFs and their target genes. TFs appeared in at least 50% of cell lines of the same phenotype (*TFBS* ‘Support Rate’ in the table is percentage of the number of TF-containing cell lines). Interestingly, the genes *CDH1*, *ESRP1* and *GRHL2* have been shown to play critical roles in epithelial-mesenchymal transition (EMT), a process associated with metastatic events in cancer and also highly relevant to tumor progression (32,33). Lombaerts *et al.* (34) reported that *CDH1* is downregulated by promoter methylation and related to EMT in breast cancer cell lines. A study by Dumont *et al.* (35) showed that the induction of EMT was accompanied by repression of *CDH1* expression and subsequent DNA hypermethylation at its promoter in basal-like breast cancer. Additionally, recent studies showed that *GRHL2* and *CDH1* in human breast cancer cells were highly correlated and suppressed EMT by repressing expression of the *ZEB1* gene (36,37). *ESRP1* was shown to regulate a switch in CD44 alternative splicing, an event required for EMT and breast cancer progression (38). Moreover, there might be potential interplay between target genes. Overexpression of *GRHL2* upregulated *ESRP1* expression (36) and *GRHL2* was shown to be essential for adequate expression of the *CDH1* and *CLDN4* (39). Thus, our approach may be useful to elucidate cell-specific regulatory mechanism using the genome-wide methylation data from the MBDCap-seq.

**Table 2.** Downregulated target gene with TFBS on hypermethylated region

Target gene	Binding TF	TFBS support rate
CDH1	SMAD1	100.0
CDH1	FOXO1	100.0
CLDN4	CEBPA	62.5
CLDN4	CEBPB	62.5
CLDN4	CEBPD	62.5
CLDN4	CEBPE	62.5
CLDN4	CEBPG	62.5
ESRP1	CUX1	90.0
GRHL2	PDX1	100.0
KRT19	PAX6	60.0
PRR15L	IKZF1	50.0
AKR1B1	E2F1	91.7
PLOD2	PAX3	100.0

## DISCUSSION

Recent developments in sequencing technologies have made it possible to analyze genome-wide DNA methylation profiles at high resolution. Although altered DNA methylation patterns are a hallmark of cancer, and promoter CGI hypermethylation is known to repress gene expression, only a few studies have examined DNA methylation-gene expression relationships using genome-wide integrated analyses (40–42). Several researchers have attempted to investigate the association of the DNA methylation with the molecular subtypes in breast cancer cells (43,44). However, high-resolution sequencing data were not used in those studies. To better understand the relationship between DNA methylation and gene expression in breast cancer molecular subtypes, we used next-generation DNA methylation sequencing data and gene expression profiles for 30 ICBP cell lines representing molecular subtypes of the disease to perform a systematic analysis.

We first compared genome-wide methylation profiles of breast cancer phenotypes. Although overall DNA methylation profiles were similar in Lu, BaA and BaB, specific genomic regions were differentially methylated among the three subtypes. We then explored computational methods for integrating DNA methylation and gene expression data and started with differentially expressed genes for discovering genes whose expressions were influenced by DNA methylation.

DNA methylation of different genomic regions has recently been associated with altered expression of downstream genes. To better understand possible transcriptional regulatory roles of DNA methylation, we performed a comprehensive study considering distinct genomic regions: CGIs, CGI shores, promoter regions, first exons, first introns and second exons. Based on Pearson’s correlation coefficients, we verified that the DNA methylation of several genomic regions including CGI and CGI shores were negatively correlated with downstream gene expression.

To investigate combinational effects of DNA methylation in these regions and to identify subtype-specific events, we applied a decision tree algorithm using genes downregulated in each subtype. Interestingly, we found potential combinatorial effects of the first exon methylation and promoter region methylation on the downstream gene expression (BaB subtype) and potential combinatorial effects of CGI methylation and CGI shore methylation (Lu subtype). As gene expression is regulated by many factors, it is difficult to predict gene expression levels using only the DNA methylation profiles. However, the classification accuracy was significantly high enough to elucidate the contribution of each genomic region and combinatorial effects of the regions. We showed that DNA methylation had combinatorial roles on gene expression, and the effects of DNA methylation in each genomic region differed among the subtypes. Moreover, our studies further imply that the aberrant DNA methylation state of the TF-associated regions could be another contributing factor to gene repression, a subject of future experimental validation.

It is now well established that different gene expression patterns contribute to breast cancer heterogeneity (22). In the current study, our integrated analysis further demonstrates that methylation status of different genomic regions may play a key role in establishing transcriptional patterns in three molecular subtypes of human breast cancer. Understanding the functional impact of distinct regions of DNA methylation on gene expression patterns may provide additional insight into breast cancer progression and response to therapy, both critical for improving management of the disease.

## SUPPLEMENTARY DATA

Supplementary Data are available at NAR Online.

## FUNDING

Next-Generation Information Computing Development Program [No. 2012M3C4A7033341]; the National Research Foundation of Korea (NRF) funded by the Ministry of Science, ICT & Future Planning (MSIP) [2012M3A9D1054622]; Next-Generation BioGreen 21 Program [PJ009037022012]; Rural Development Administration, Republic of Korea; This work is also part of National Cancer Institute, Integrated Cancer Biology Program, Center for Cancer Systems Biology, funded by [U54 CA113001] (Interrogating Epigenetic Changes in Cancer Genomes); NRF grant funded by MSIP [No. 2010-0017734 to J.K.R and B.T.Z.] (in part). Funding for open access charge: Seoul National University.

*Conflict of interest statement*. None declared.

## Supplementary Material

Supplementary Data

## References

[1] Suzuki M, Bird A (2008). DNA methylation landscapes: provocative insights from epigenomics. Nat. Rev. Genet..

[2] Robertson K (2005). DNA methylation and human disease. Nat. Rev. Genet..

[3] Esteller M (2008). Epigenetics in cancer. N. Engl. J. Med..

[4] Keshet I, Schlesinger Y, Farkash S, Rand E, Hecht M, Segal E, Pikarski E, Young RA, Niveleau A, Cedar H (2006). Evidence for an instructive mechanism of de novo methylation in cancer cells. Nat. Genet..

[5] Jones PA, Takai D (2001). The role of DNA methylation in mammalian epigenetics. Science.

[6] Ueki T, Walter KM, Skinner H, Jaffee E, Hruban RH, Goggins M (2002). Aberrant CpG island methylation in cancer cell lines arises in the primary cancers from which they were derived. Oncogene.

[7] Sakai T, Toguchida J, Ohtani N, Yandell DW, Rapaport JM, Dryja TP (1991). Allele-specific hypermethylation of the retinoblastoma tumor-suppressor gene. Am. J. Hum. Genet..

[8] Merlo A, Herman JG, Mao L, Lee DJ, Gabrielson E, Burger PC, Baylin SB, Sidransky D (1995). 5′ CpG island methylation is associated with transcriptional silencing of the tumour suppressor p16/CDKN2/MTS1 in human cancers. Nat. Med..

[9] Chavez L, Jozefczuk J, Grimm C, Dietrich J, Timmermann B, Lehrach H, Herwig R, Adjaye J (2010). Computational analysis of genome-wide DNA methylation during the differentiation of human embryonic stem cells along the endodermal lineage. Genome Res..

[10] Kim JH, Dhanasekaran SM, Prensner JR, Cao X, Robinson D, Kalyana-Sundaram S, Huang C, Shankar S, Jing X, Iyer M (2011). Deep sequencing reveals distinct patterns of DNA methylation in prostate cancer. Genome Res..

[11] Zuo T, Tycok B, Liu TM, Lin HJ, Huang TH (2009). Methods in DNA methylation profiling. Epigenomics.

[12] Rauch TA, Pfeifer GP (2010). DNA methylation profiling using the methylated-CpG island recovery assay (MIRA). Methods.

[13] Brinkman AB, Simmer F, Ma K, Kaan A, Zhu J, Stunnenberg HG (2010). Whole-genome DNA methylation profiling using MethylCap-seq. Methods.

[14] Robinson MD, Stirzaker C, Statham AL, Coolen MW, Song JZ, Nair SS, Strbenac D, Speed TP, Clark SJ (2010). Evaluation of affinity-based genome-wide DNA methylation data: effects of CpG density, amplification bias, and copy number variation. Genome Res..

[15] Esteller M (2007). Epigenetic gene silencing in cancer: the DNA hypermethylome. Hum. Mol. Genet..

[16] Bell JT, Pai AA, Pickrell JK, Gaffney DJ, Pique-Regi R, Degner JF, Gilad Y, Pritchard JK (2011). DNA methylation patterns associate with genetic and gene expression variation in HapMap cell lines. Genome Biol..

[17] Pai AA, Bell JT, Marioni JC, Pritchard JK, Gilad Y (2011). A genome-wide study of DNA methylation patterns and gene expression levels in multiple human and chimpanzee tissues. PLoS Genet..

[18] Sproul D, Nestor C, Culley J, Dickson JH, Dixon JM, Harrison DJ, Meehan RR, Sims AH, Ramsahoye BH (2011). Transcriptionally repressed genes become aberrantly methylated and distinguish tumors of different lineages in breast cancer. Proc. Natl Acad. Sci. USA.

[19] Irizarry RA, Ladd-Acosta C, Wen B, Wu Z, Montano C, Onyango P, Cui H, Gabo K, Rongione M, Webster M (2009). The human colon cancer methylome shows similar hypo- and hypermethylation at conserved tissue-specific CpG island shores. Nat. Genet..

[20] Brenet F, Moh M, Funk P, Feierstein E, Viale AJ, Socci ND, Scandura JM (2011). DNA methylation of the first exon is tightly linked to transcriptional silencing. PLoS One.

[21] Neve RM, Chin K, Fridlyand J, Yeh J, Baehner FL, Fevr T, Clark L, Bayani N, Coppe JP, Tong F (2006). A collection of breast cancer cell lines for the study of functionally distinct cancer subtypes. Cancer Cell.

[22] Cancer Genome Atlas Network (2012). Comprehensive molecular portraits of human breast tumours. Nature.

[23] Fadare O, Tavassoli FA (2008). Clinical and pathologic aspects of basal-like breast cancers *Nat*. Clin. Pract. Oncol..

[24] Toft DJ, Cryns VL (2011). Minireview: Basal-like breast cancer: from molecular profiles to targeted therapies. Mol. Endocrinol..

[25] Rao X, Evans J, Chae H, Pilrose J, Kim S, Yan P, Huang RL, Lai HC, Lin H, Liu Y (2012). CpG island shore methylation regulates caveolin-1 expression in breast cancer. Oncogene.

[26] Hall M, Frank E, Holmes G, Pfahringer B, Reutemann P, Witten IH (2009). The WEKA data mining software: an update. SIGKDD Explor..

[27] Matys V, Kel-Margoulis OV, Fricke E, Liebich I, Land S, Barre-Dirrie A, Reuter I, Chekmenev D, Krull M, Hornischer K (2006). TRANSFAC and its module TRANSCompel: transcriptional gene regulation in eukaryotes. Nucleic Acids Res..

[28] Kel AE, Gössling E, Reuter I, Cheremushkin E, Kel-Margoulis OV, Wingender E (2003). MATCH: A tool for searching transcription factor binding sites in DNA sequences. Nucleic Acids Res..

[29] Takai D, Jones PA (2002). Comprehensive analysis of CpG islands in human chromosomes 21 and 22. Proc. Natl Acad. Sci. USA.

[30] Sloan EK, Stanley KL, Anderson RL (2004). Caveolin-1 inhibits breast cancer growth and metastasis. Oncogene.

[31] Savage K, Lambros MB, Robertson D, Jones RL, Jones C, Mackay A, James M, Hornick JL, Pereira EM, Milanezi F (2007). Caveolin 1 is overexpressed and amplified in a subset of basal-like and metaplastic breast carcinomas: a morphologic, ultrastructural, immunohistochemical, and in situ hybridization analysis. Clin. Cancer Res..

[32] Thiery JP (2002). Epithelial-mesenchymal transitions in tumour progression. Nat. Rev. Cancer.

[33] Thiery JP, Acloque H, Huang RYJ, Nieto MA (2009). Epithelial-mesenchymal transitions in development and disease. Cell.

[34] Lombaerts M, van Wezel T, Philippo K, Dierssen JWF, Zimmerman RME, Oosting J, van Eijk R, Eilers PH, van de Water B, Cornelisse CJ (2006). E-cadherin transcriptional downregulation by promoter methylation but not mutation is related to epithelial-to-mesenchymal transition in breast cancer cell lines. Br. J. Cancer.

[35] Dumont N, Wilson MB, Crawford YG, Reynolds PA, Sigaroudinia M, Tlsty TD (2008). Sustained induction of epithelial to mesenchymal transition activates DNA methylation of genes silenced in basal-like breast cancers. Proc. Natl Acad. Sci. USA.

[36] Xiang X, Deng ZB, Zhuang X, Ju S, Mu J, Jiang H, Zhang L, Yan J, Miller D, Zhang HG (2012). Grhl2 determines the epithelial phenotype of breast cancers and promotes tumor progression. PloS One.

[37] Cieply B, Riley P, Pifer PM, Widmeyer J, Addison JB, Ivanov AV, Denvir J, Frisch SM (2012). Suppression of the epithelial-mesenchymal transition by grainyhead-like-2. Cancer Res..

[38] Brown RL, Reinke LM, Damerow MS, Perez D, Chodosh LA, Yang J, Cheng C (2011). CD44 splice isoform switching in human and mouse epithelium is essential for epithelial-mesenchymal transition and breast cancer progression. J. Clin. Invest..

[39] Werth M, Walentin K, Aue A, Schönheit J, Wuebken A, Pode-Shakked N, Vilianovitch L, Erdmann B, Dekel B, Bader M (2010). The transcription factor grainyhead-like 2 regulates the molecular composition of the epithelial apical junctional complex. Development.

[40] Ruike Y, Imanaka Y, Sato F, Shimizu K, Tsujimoto G (2010). Genome-wide analysis of aberrant methylation in human breast cancer cells using methyl-DNA immunoprecipitation combined with high-throughput sequencing. BMC Genomics.

[41] Fang F, Turcan S, Rimner A, Kaufman A, Giri D, Morris LG, Shen R, Seshan V, Mo Q, Heguy A (2011). Breast cancer methylomes establish an epigenomic foundation for metastasis. Sci. Transl. Med..

[42] Sun Z, Asmann YW, Kalari KR, Bot B, Eckel-Passow JE, Baker TR, Carr JM, Khrebtukova I, Luo S, Zhang L (2011). Integrated analysis of gene expression, CpG island methylation, and gene copy number in breast cancer cells by deep sequencing. PLoS One.

[43] Bloushtain-Qimron N, Yao J, Snyder EL, Shipitsin M, Campbell LL, Mani SA, Hu M, Chen H, Ustyansky V, Antosiewicz JE (2008). Cell type-specific DNA methylation patterns in the human breast. Proc. Natl Acad. Sci. USA.

[44] Holm K, Hegardt C, Staaf J, Vallon-Christersson J, Jonsson G, Olsson H, Borg A, Ringner M (2010). Molecular subtypes of breast cancer are associated with characteristic DNA methylation patterns. Breast Cancer Res..

