# Supplementary material for: Integrated analysis of genome-wide DNA methylation and gene expression profiles in molecular subtypes of breast cancer
Source: Nucleic Acids Res. 2013 Jul 24;41(18):8464–74. doi: 10.1093/nar/gkt643 (PMC3794600; doi:10.1093/nar/gkt643)
Supplement: Supplementary Data [file supp_41_18_8464__index.html]

Integrated analysis of genome-wide DNA methylation and gene expression profiles in molecular subtypes of breast cancer — Integrated analysis of genome-wide DNA methylation and gene expression profiles in molecular subtypes of breast cancer — Supplementary Data 

# Integrated analysis of genome-wide DNA methylation and gene expression profiles in molecular subtypes of breast cancer

## 

files

**Files in this Data Supplement:**

- Supplementary Data - pdf file
- Supplementary Data - pdf file
