# Supplementary material for: Integrated analysis of genome-wide DNA methylation and gene expression profiles in molecular subtypes of breast cancer
Source: Nucleic Acids Res. 2013 Jul 24;41(18):8464–74. doi: 10.1093/nar/gkt643 (PMC3794600; doi:10.1093/nar/gkt643)
Supplement: Supplementary Data [file supp_gkt643_nar-00917-n-2013-File001.pdf]

Genome-wide Integrated Analysis of Genome-wide DNA Methylation and Gene Expression Profiles in Breast Cancer Molecular Subtypes of Breast Cancer

SUPPLEMENTARY DATA

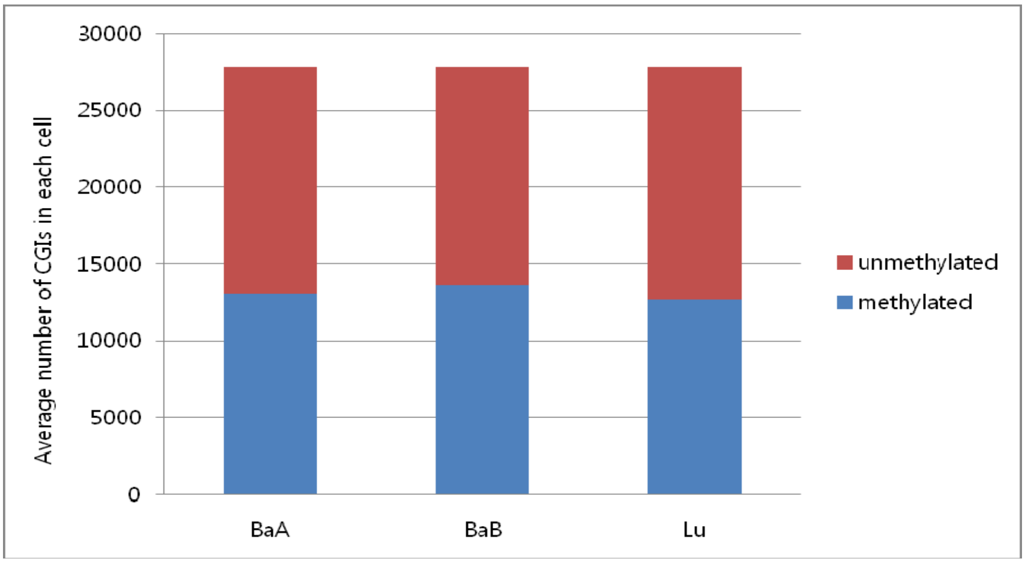

Supplementary Figure 1. Average number of methylated and unmethylated CGIs in each cell. BaA, basal A; BaB, basal B; Lu, luminal.

Supplementary Table 1. Genes that were both differentially methylated and expressed.

Gene Name	Description
**PLA2G12A**	**phospholipase A2, group XIIA**
**FAT1**	**FAT tumor suppressor homolog 1**
**PARP8**	**poly (ADP-ribose) polymerase family, member 8**
