# Supplementary material for: Integrated analysis of genome-wide DNA methylation and gene expression profiles in molecular subtypes of breast cancer
Source: Nucleic Acids Res. 2013 Jul 24;41(18):8464–74. doi: 10.1093/nar/gkt643 (PMC3794600; doi:10.1093/nar/gkt643)
Supplement: Supplementary Data [file supp_gkt643_nar-00917-n-2013-File014.pdf]

Integrated Analysis of Genome-wide DNA Methylation and Gene Expression Profiles in Breast Cancer Molecular Subtypes of Breast Cancer

SUPPLEMENTARY DATA

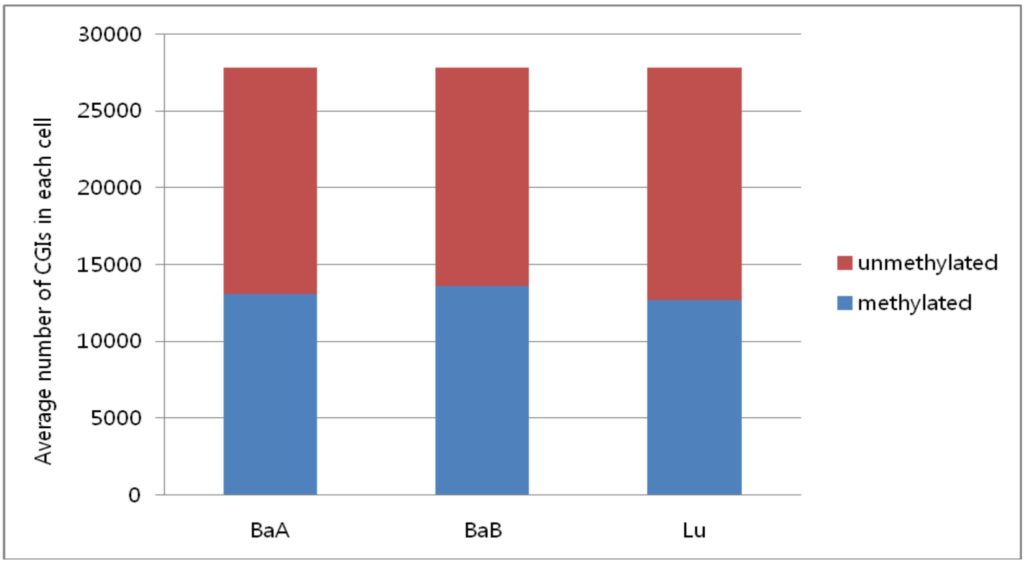

Supplementary Figure 1. Average number of methylated and unmethylated CGIs in each cell. The unmethylated means that the mapped read count is zero in the CGI. BaA: basal A, BaB: basal B, Lu: luminal.

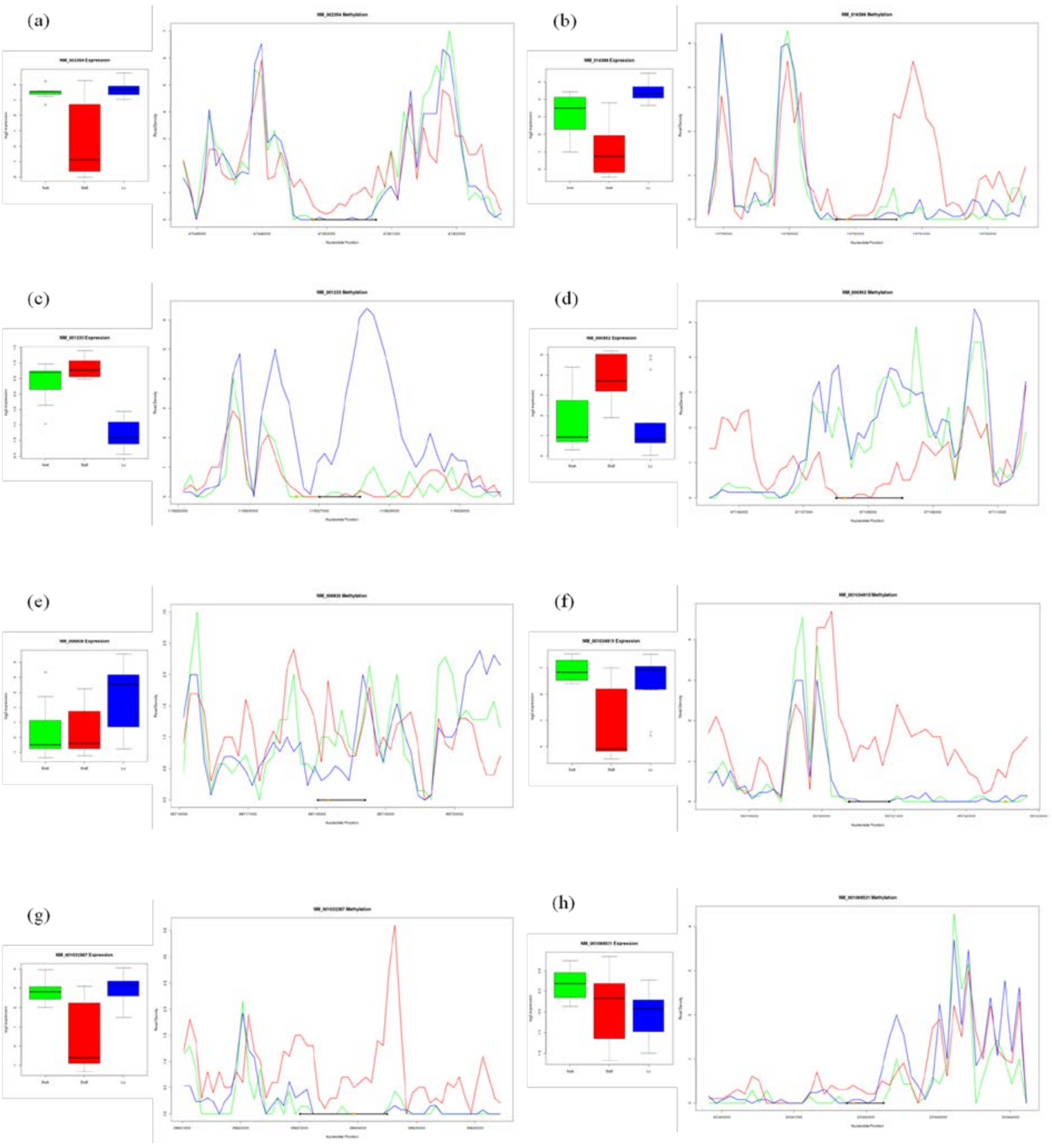

Supplementary Figure 2. Examples of CGI methylation and gene expression. The genes only on positive strand were presented for consistent direction in the plots. Lu; green, BaA; red and BaB; blue. (a) NM_002354 (The correlation coefficient ρ (between the CGI methylation and the gene expression) is -0.881), (b) NM_014399 (ρ=-0.839), (c) NM_001233 (ρ=-0.800), (d) NM_000852 (ρ=-0.785), (e) NM_006829 (ρ=-0.763), (f) NM_001034915 (ρ=-0.760), (g) NM_001032367 (ρ=-0.759) and (h) NM_001098521 (ρ=-0.749).

(a)

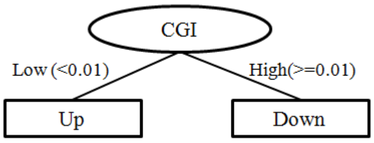
CGI
Low (<0.01)
High(>=0.01)
Up
Down

(b)

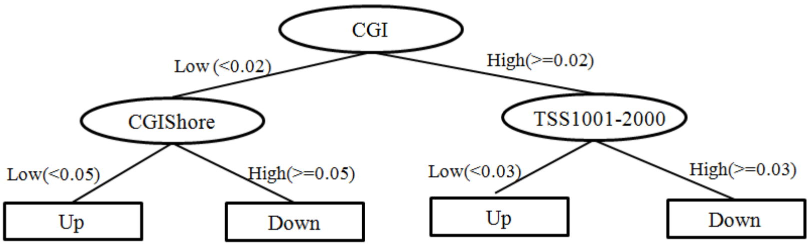
CGI
Low (<0.02)
High(>=0.02)
CGIShore
TSS1001-2000
Low(<0.05)
High(>=0.05)
Low(<0.03)
High(>=0.03)
Up
Down
Up
Down

(c)

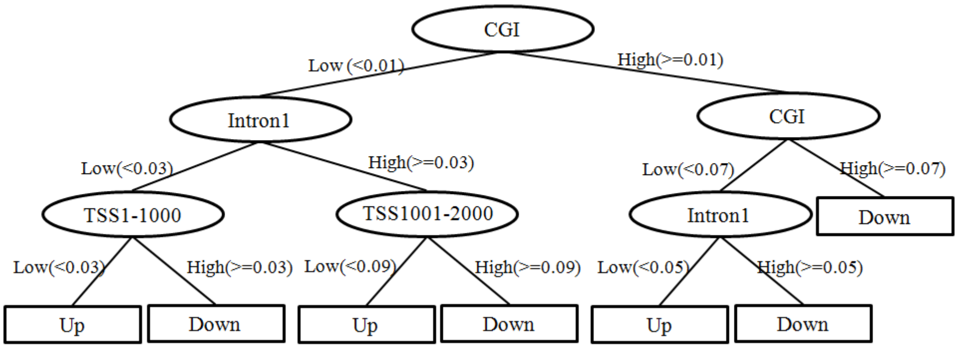
CGI
Low (<0.01)
High(>=0.01)
Intron1
CGI
Low(<0.03)
High(>=0.03)
Low(<0.07)
High(>=0.07)
TSS1-1000
TSS1001-2000
Intron1
Down
Low(<0.03)
High(>=0.03)
Low(<0.09)
High(>=0.09)
Low(<0.05)
High(>=0.05)
Up
Down
Up
Down
Up
Down

(d)

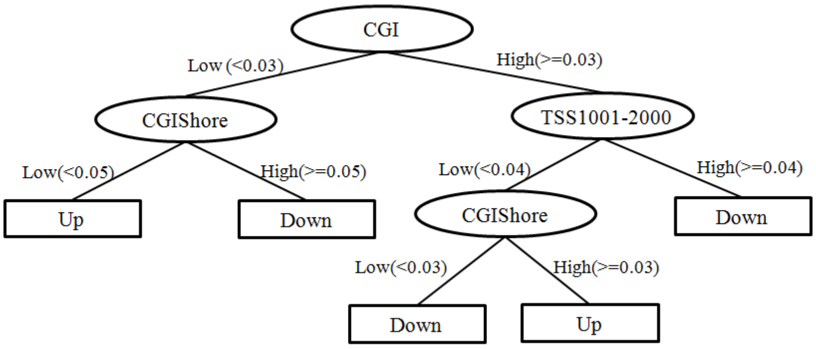
CGI
Low (<0.03)
High(>=0.03)
CGIShore
TSS1001-2000
Low(<0.05)
High(>=0.05)
Low(<0.04)
High(>=0.04)
Up
Down
CGIShore
Down
Low(<0.03)
High(>=0.03)
Down
Up

(e)

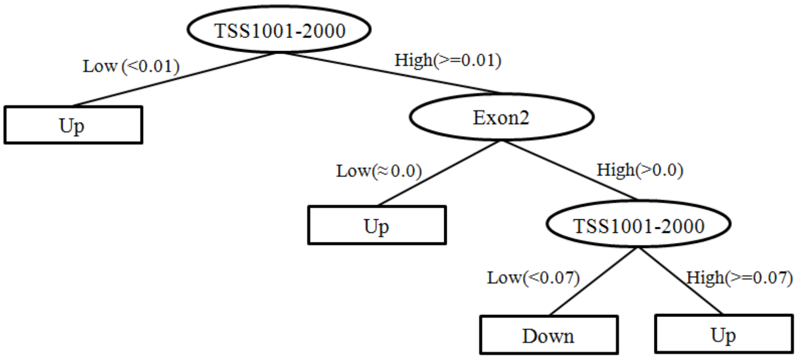
TSS1001-2000
Low (<0.01)
High(>=0.01)
Up
Exon2
Low(≈0.0)
High(>0.0)
Up
TSS1001-2000
Low(<0.07)
High(>=0.07)
Down
Up

(f)

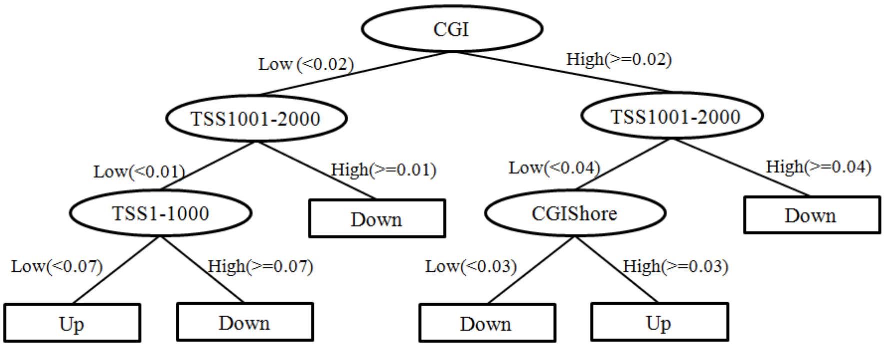
CGI
Low (<0.02)
High(>=0.02)
TSS1001-2000
TSS1001-2000
Low(<0.01)
High(>=0.01)
Low(<0.04)
High(>=0.04)
TSS1-1000
Down
CGIShore
Down
Low(<0.07)
High(>=0.07)
Low(<0.03)
High(>=0.03)
Up
Down
Down
Up

Supplementary Figure 3. In case of down-regulation in Lu subtype, decision tree analysis separated by genomic regions of CGI. (a) Overlap with the first exon (The classification accuracy, Acc. is 0.737), (b) Nonoverlap with the first exon (Acc. is 0.590),(c) Overlap with TSS1-1000 (Acc. is 0.687), (d) Nonoverlap with TSS1-1000 (Acc. is 0.644), (e) Overlap with TSS1001-2000 (Acc. is 0.644) and (f) Nonoverlap with TSS1001-2000 (Acc. is 0.644).

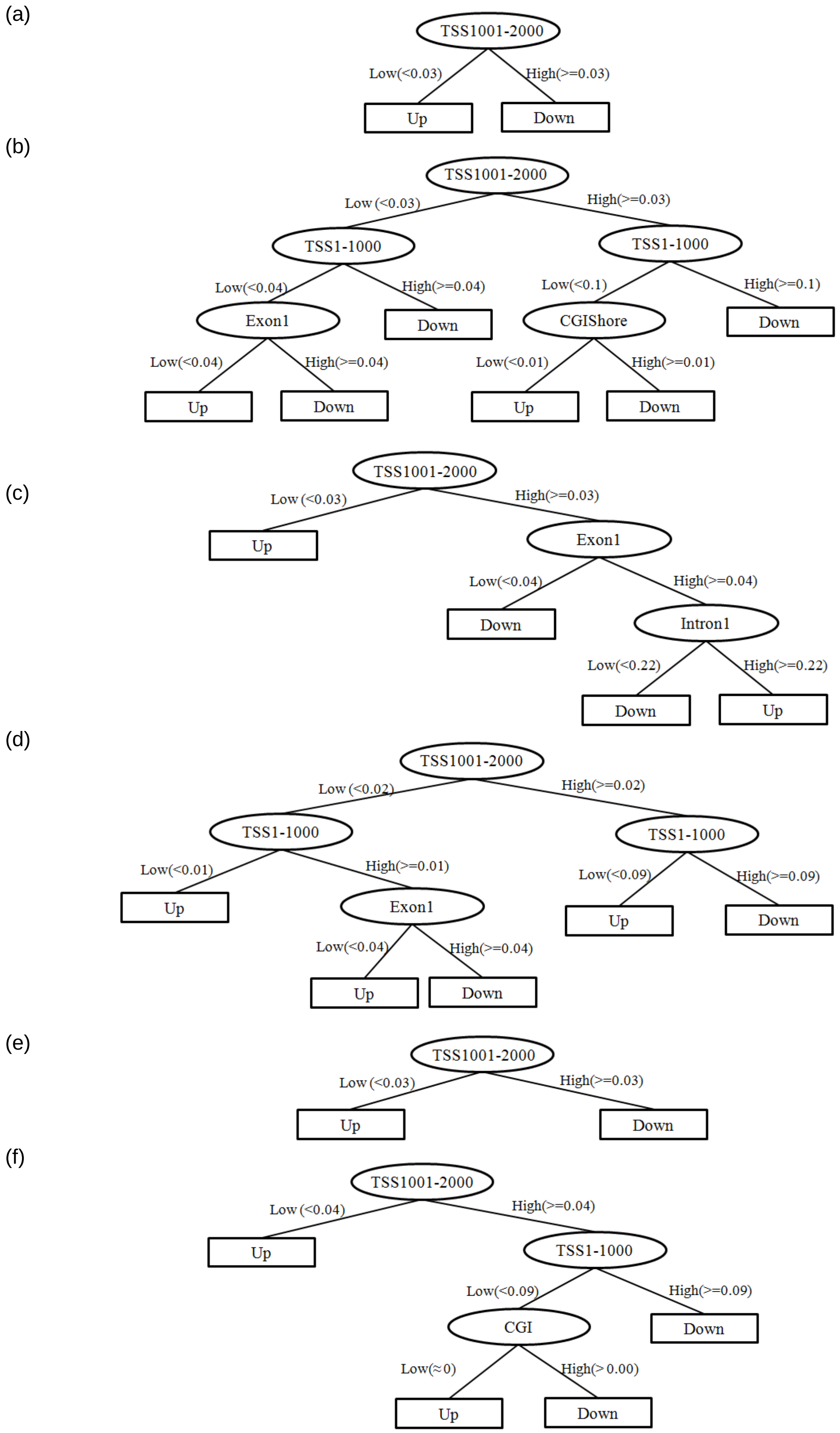
(a)
TSS1001-2000
Low(<0.03)
High(>=0.03)
Up
Down
(b)
TSS1001-2000
Low (<0.03)
High(>=0.03)
TSS1-1000
TSS1-1000
Low(<0.04)
High(>=0.04)
Low(<0.1)
High(>=0.1)
Exon1
Down
CGIShore
Down
Low(<0.04)
High(>=0.04)
Low(<0.01)
High(>=0.01)
Up
Down
Up
Down
(c)
TSS1001-2000
Low (<0.03)
High(>=0.03)
Up
Exon1
Low(<0.04)
High(>=0.04)
Down
Intron1
Low(<0.22)
High(>=0.22)
Down
Up
(d)
TSS1001-2000
Low (<0.02)
High(>=0.02)
TSS1-1000
TSS1-1000
Low(<0.01)
High(>=0.01)
Low(<0.09)
High(>=0.09)
Up
Exon1
Up
Down
Low(<0.04)
High(>=0.04)
Up
Down
(e)
TSS1001-2000
Low (<0.03)
High(>=0.03)
Up
Down
(f)
TSS1001-2000
Low (<0.04)
High(>=0.04)
Up
TSS1-1000
Low(<0.09)
High(>=0.09)
CGI
Down
Low(≈0)
High(> 0.00)
Up
Down

Supplementary Figure 4. In case of down-regulation in BaB subtype, decision tree analysis separated by genomic regions of CGI. (a) Overlap with the first exon (Acc. is 0.773), (b) Nonoverlap with the first exon (Acc. is 0.760),(c) Overlap with TSS1-1000 (Acc. is 0.810), (d) Nonoverlap with TSS1-1000 (Acc. is 0.708), (e) Overlap with TSS1001-2000 (Acc. is 0.824) and (f) Nonoverlap with TSS1001-2000 (Acc. is 0.741).

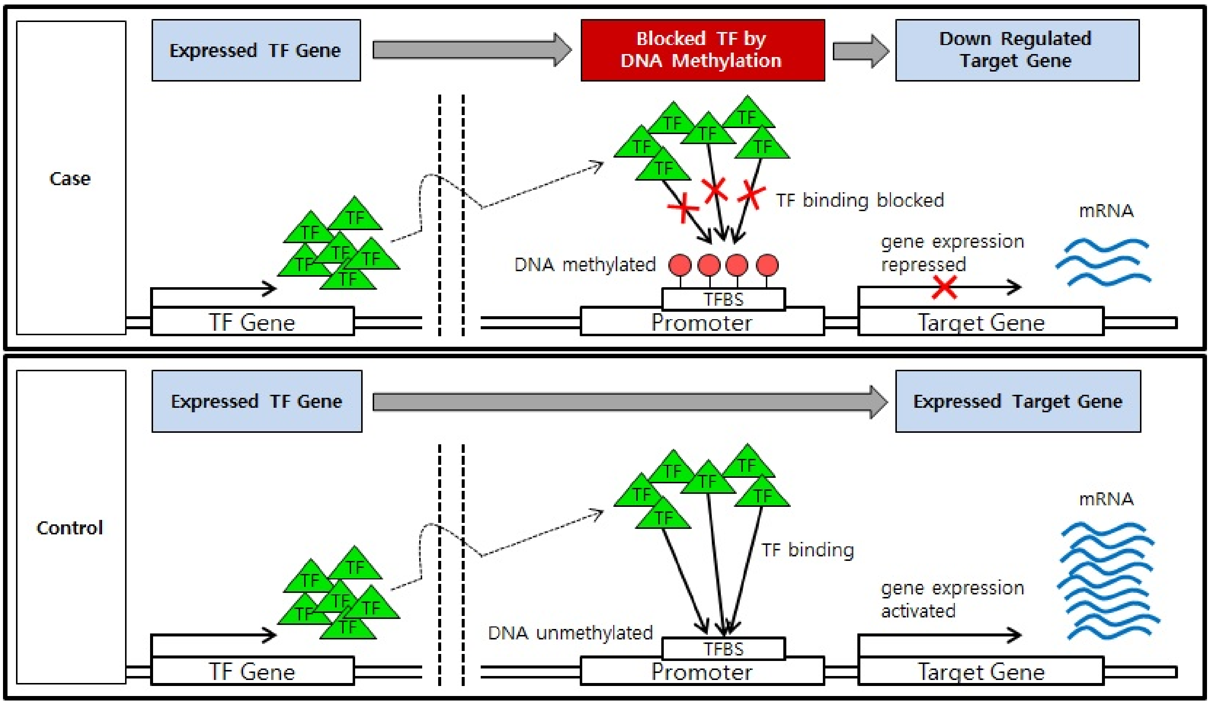

Supplementary Figure 5. Schematic overview of the phenotype-comparative analysis for interference of TF binding by DNA methylation resulting in the suppression of downstream gene expression.

Supplementary Table 1. Genes that were both differentially methylated and expressed.

Gene Name	Description
**PLA2G12A**	**phospholipase A2, group XIIA**
**FAT1**	**FAT tumor suppressor homolog 1**
**PARP8**	**poly (ADP-ribose) polymerase family, member 8**

Supplementary Table 2. Genes presented in Figure 5 (a)

Refseq ID	Gene Name
NM_016303	WBP5
NM_001136025	PLS3
NM_002444	MSN
NM_001233	CAV2
NM_001001390	CD44
NM_018004	TMEM45A
NM_022763	FNDC3B
NM_001753	CAV1
NM_001079818	ITGA6
NM_144779	FXYD5
NM_006359	SLC9A6
NR_028406	
NM_000676	ADORA2B
NM_001444	FABP5
NM_001353	AKR1C1
NM_001164605	FXYD5
NM_003739	AKR1C3
NM_001161574	MAFF
NM_021154	PSAT1
NM_001111097	LYN
NM_032525	TUBB6
NM_006435	IFITM2
NM_005363	MAGEA6
NM_002305	LGALS1
NM_001924	GADD45A
NM_003380	VIM
NM_001172896	CAV1
NM_001172897	CAV1
NM_001172895	CAV1
NM_005032	PLS3
NM_001135095	FNDC3B
NM_001177651	SLC9A6
NM_005531	IFI16
NM_000700	ANXA1
NM_001161573	MAFF
NM_014164	FXYD5

Supplementary Table 3. Genes presented in Figure 5 (b)

Refseq ID	Gene Name
NM_004448	ERBB2
NM_001122826	ESRP1
NM_003710	SPINT1
NM_021910	FXYD3
NM_015570	AUTS2
NM_001305	CLDN4
NM_001112719	LIMCH1
NM_001112718	LIMCH1
NM_001112717	LIMCH1
NM_014399	TSPAN13
NM_001003396	TPD52L1
NM_001003395	TPD52L1
NM_005853	IRX5
NM_004433	ELF3
NM_001769	CD9
NM_021102	SPINT2
NM_001008844	DSP
NM_012342	BAMBI
NM_014988	LIMCH1
NM_001127231	AUTS2
NM_002354	EPCAM
NM_001677	ATP1B1
NM_002193	INHBB
NM_000224	KRT18
NM_001954	DDR1
NM_205835	LSR
NM_001005862	ERBB2
NM_002483	CEACAM6
NM_030777	SLC2A10
NM_201525	GPR56
NM_199187	KRT18
NM_020387	RAB25
NM_005682	GPR56
NM_013994	DDR1
NM_013993	DDR1
NM_014298	QPRT
NM_004360	CDH1
NM_001032367	SPINT1
NM_001145770	GPR56
NM_001145774	GPR56
NM_002585	PBX1
NM_000597	IGFBP2
NM_024915	GRHL2
NM_005980	S100P

Supplementary Table 4. P-value for correlation coefficients between promoter methylation and gene expression of down-regulated genes in Lu and BaB

Distance from TSS (bp)	p-value (t-test)
1-100	0.309
101-200	0.469
201-300	0.06
301-400	0.058
401-500	0.029
501-600	0.046
601-700	0.205
701-800	0.179
801-900	0.049
901-1000	0.046
1001-1100	0.010
1101-1200	4.59E-05
1201-1300	2.26E-04
1301-1400	6.12E-04
1401-1500	1.82E-04
1501-1600	2.32E-04
1601-1700	0.001
1701-1800	0.042
1801-1900	0.002
1901-2000	0.107

Supplementary Table 5. Genes presented in Figure 6 (a)

Refseq ID	Gene Name
NM_014164	FXYD5
NM_175868	MAGEA6
NM_001143678	SGK1
NM_001143677	SGK1
NM_017611	SLC43A3
NM_001161572	MAFF
NM_001161573	MAFF
NM_001136528	SERPINE2
NM_005627	SGK1
NM_005842	SPRY2
NM_001177651	SLC9A6
NM_182943	PLOD2
NM_198212	CAV2
NM_006216	SERPINE2
NM_001135095	FNDC3B
NM_005032	PLS3
NM_001172895	CAV1
NM_001172897	CAV1
NM_001172896	CAV1
NM_012323	MAFF
NM_001042537	SLC9A6
NM_004159	PSMB8
NM_003380	VIM
NM_001924	GADD45A
NM_148919	PSMB8
NM_002305	LGALS1
NM_000581	GPX1
NM_005362	MAGEA3
NM_005363	MAGEA6
NM_018192	LEPREL1
NM_001136530	SERPINE2
NM_006435	IFITM2
NR_024539	
NM_032525	TUBB6
NM_001111097	LYN
NM_001628	AKR1B1
NM_001143676	SGK1
NM_000228	LAMB3
NM_021154	PSAT1
NM_006548	IGF2BP2
NM_001161574	MAFF
NM_014096	SLC43A3
NM_199329	SLC43A3
NM_001134418	LEPREL1
NM_024745	SHCBP1
NM_016205	PDGFC
NM_001164605	FXYD5

NM_001444	FABP5
NM_000676	ADORA2B
NR_028406	
NM_006359	SLC9A6
NM_000214	JAG1
NM_006681	NMU
NM_007085	FSTL1
NM_002061	GCLM
NM_144779	FXYD5
NM_001127641	LAMB3
NM_001079818	ITGA6
NM_003068	SNAI2
NM_001753	CAV1
NM_022763	FNDC3B
NM_021034	IFITM3
NM_018004	TMEM45A
NM_201397	GPX1
NM_022361	POPDC3
NM_001001390	CD44
NM_001013398	IGFBP3
NM_001233	CAV2
NM_002444	MSN
NM_001017402	LAMB3
NM_001178096	F3

Supplementary Table 6. Genes presented in Figure 6 (b)

Refseq ID	Gene Name
NM_014164	FXYD5
NM_024915	GRHL2
NM_000597	IGFBP2
NM_005749	TOB1
NM_002275	KRT15
NM_002276	KRT19
NM_002585	PBX1
NM_001166103	SPINT2
NM_001032367	SPINT1
NM_004360	CDH1
NM_014298	QPRT
NM_013993	DDR1
NM_013994	DDR1
NM_019027	RBM47
NM_018728	MYO5C
NM_020387	RAB25
NM_013230	
NM_001017970	TMEM30B
NM_199187	KRT18
NM_002773	PRSS8
NM_015677	SH3YL1
NM_030777	SLC2A10
NM_001005862	ERBB2
NM_002353	TACSTD2
NM_003355	UCP2
NM_205835	LSR
NM_002705	PPL
NM_004335	BST2
NM_001954	DDR1
NM_000224	KRT18
NM_002193	INHBB
NM_001185022	CLDN7
NM_001677	ATP1B1
NM_002354	EPCAM
NM_001127231	AUTS2
NM_014988	LIMCH1
NM_012342	BAMBI
NM_001008844	DSP
NM_021102	SPINT2
NM_002281	KRT81
NM_001769	CD9
NM_001878	CRABP2
NM_005853	IRX5
NM_001003395	TPD52L1
NM_001003396	TPD52L1
NM_014399	TSPAN13
NM_001112717	LIMCH1

NM_001112718	LIMCH1
NM_001112719	LIMCH1
NM_007365	PADI2
NM_002273	KRT8
NM_001305	CLDN4
NM_001307	CLDN7
NM_021991	JUP
NM_015570	AUTS2
NM_021910	FXYD3
NM_007210	GALNT6
NM_003710	SPINT1
NM_001122826	ESRP1
NM_001098634	RBM47
NM_004669	CLIC3
NM_004448	ERBB2
NM_018584	CAMK2N1
